# An Immunogenic Cell Death-Related Gene Signature Reflects Immune Landscape and Predicts Prognosis in Melanoma Independently of BRAF V600E Status

**DOI:** 10.1155/2023/1189022

**Published:** 2023-01-16

**Authors:** Lizhu Chen, Yaoming Wen, Jiani Xiong, Yu Chen, Chuan-ben Chen

**Affiliations:** ^1^Department of Medical Oncology, Clinical Oncology School of Fujian Medical University, Fujian Cancer Hospital, Fuzhou, Fujian Province, China; ^2^Fujian institute of microbiology, Fuzhou, Fujian Province, China; ^3^Department of Radiation Oncology, Clinical Oncology School of Fujian Medical University, Fujian Cancer Hospital, Fuzhou, Fujian Province, China

## Abstract

Immunogenic cell death (ICD) is a type of regulated cell death that can activate adaptive immune response, and its ability to reshape the tumor microenvironment via multiple mechanisms may contribute to immunotherapy. The treatment options for patients with skin cutaneous melanoma (SKCM) vary based on BRAF V600E statuses. However, all standard treatments include immunotherapy. Therefore, it is critical to identify ICD-associated signatures that can help classify patients according to benefits from ICD immunotherapy. In this study, data on melanoma samples with BRAF V600E mutation (BRAF V600E-mutant melanoma) and melanoma samples with wild-type BRAF V600E alleles (BRAF V600E WT melanoma) were collected from The Cancer Genome Atlas (TCGA) database. The ICD-related (ICD-high and ICD-low) subgroups of patients with BRAF V600E WT melanoma were established via consensus clustering. The analyses of survival, differentially expressed genes (DEGs), functional annotation, and immune landscape were performed in these two subgroups. Results showed that ICD-high subgroup was correlated with a positive overall survival (OS) and active tumor immune landscape. A model comprising seven prognosis ICD-related gene biomarkers was developed. Survival analysis and receiver operating characteristic (ROC) curve evaluation in both cohorts with BRAF V600E WT and BRAF V600E-mutant melanoma showed an accurate prognostic estimation of ICD-related risk signature. There was a correlation between immune cell infiltration and immunotherapy response and risk score. Thus, the ICD risk signature was closely associated with the tumor's immune microenvironment. Our results may provide insights to further individualize and improve precision therapeutic decision-making in BRAF V600E-mutant and WT melanoma.

## 1. Introduction

Melanoma is a malignant tumor of the epidermis caused by the uncontrolled division of melanocytes [1]. The 5-year survival rates were 97%, 84%, 68%, 55%, and 17% in patients with stage I, II, III, and IV cutaneous melanoma (CM), respectively [2]. Therefore, early detection and the use of predictive biomarkers for prognosis are essential to improve the survival of patients with CM who have a poor prognosis.

The B-Raf proto-oncogene serine/threonine-kinase (BRAF), which is located on chromosome 7q34, regulates the mitogen-activated protein kinase/extracellular signal-regulated kinase signaling pathway. BRAF plays a role in the growth, proliferation, invasion, and metastasis of tumor cells [3]. Approximately 50% of melanomas contain activating point mutations in the BRAF gene, with the most common V600E mutation [4]. Treatments with combined selected BRAF inhibitors and mitogen-activated protein kinase inhibitors have significantly improved response and overall survival in patients with BRAF V600-mutant melanoma. However, less than half of patients present with long-term benefits [5–7].

Immunotherapy via the application of antibody inhibitors programmed cell death protein-1 (PD1)/programmed death ligand-1 (PDL1) has been a great breakthrough in the treatment of cutaneous melanoma [8–10]. However, the therapeutic efficiency of immunotherapy in melanoma should be improved [10, 11]. Therefore, the efficacy of combined BRAF inhibitor-based targeted therapy and immune checkpoint blockade must be further evaluated [12]. Preclinical [13–15] and clinical [16, 17] studies have found that a combined treatment strategy can affect the tumor's immune microenvironment and promote antitumor effects in melanoma. A previous report revealed that patient stratification and selection significantly improved with the use of biomarkers reflecting the immune microenvironment, or models that can predict the efficacy of immunotherapy [18]. It is essential to identify biomarkers and develop risk models for predicting the immune microenvironment, prognosis, and immunotherapy response in BRAF V600E-mutant and WT melanoma.

ICD is a type of regulated cell death that can activate an adaptive immune response in immunocompetent syngeneic hosts [19, 20]. Damage-associated molecular patterns are mainly responsible for the immunogenic characteristics of ICD, which can be recognized by pattern recognition receptors that induce the activation, differentiation, and maturation of antigen-presenting cells [21]. ICD can trigger antitumor immunity, which attracts immune-effector cells to the tumor microenvironment and inhibits the development of immune system-dependent tumors, thereby improving survival of the host [22–24]. Therefore, the ability of ICD in activating anticancer immune responses is evaluated more closely [25].

The commonly induced stresses of ICD include tumor viruses and physiotherapy [26]. Various stresses can enhance ICD in melanoma, such as reactive oxygen species-triggered nanoinducer based on dermatan sulfate [27], zinc oxide-based multifunctional nanocomposites [28], and combined novel calreticulin-nanoparticles and focused ultrasonography [29]. In a mouse melanoma model, immune cells accumulate, and they are activated with a higher ICD [30]. The cold tumor microenvironment transformed to hot, thereby improving response to antiprogrammed cell death proteins and promoting tumor regression. The use of risk prediction models for the precision immune treatment of patients with BRAF V600E WT and BRAF V600E-mutant skin cutaneous melanoma (SKCM) is an issue worth exploring. It is reported that ICD-related genes (IRGs) are associated with improved survival of patients with lung, breast, or ovarian malignancies [31]. In this report, the ICD parameters were evaluated via a comprehensive literature review (Web of Knowledge, Scopus, and PubMed for pertinent research investigations performed in vivo using mice and/or in vitro using primary human immune cells). But roles of IRGs in melanoma independently of BRAF V600E status are still unknown. Therefore, the current study is aimed at stratifying IRGs to evaluate prognosis and immunotherapy response in patients with BRAF V600E WT and BRAF V600E-mutant melanoma.

## 2. Methods

### 2.1. Data Extraction

Data on the transcriptome profiling and clinical characteristics of 470 patients with melanoma were collected from TCGA (https://portal.gdc.cancer.gov/) (Table S1). The cohort with BRAF V600E WT melanoma comprised 278 patients. Meanwhile, the cohort with BRAF V600E-mutant melanoma included 192 patients. Using the Perl programming language (version Strawberry-Perl-5.30.0; https://www.perl.org), the RNA-seq data were extracted in the fragment per kilobase million format. Simultaneously, clinical data were preprocessed with Pearl to obtain the complete pathological information of clinical samples.

### 2.2. Consensus Clustering

In total, 34 ICD-related genes were selected for further analysis according to the previous study [31]. Unsupervised class discovery is useful for identifying specific common biological features between the existing classification systems [32]. Consensus clustering enables quantification and visualization for calculating the number of unsupervised classes in a database [33]. After 1000 repetitions, an ideal number of clusters was determined (*k* = 2–10) to identify subtypes associated with ICD using the Limma and ConsensusClusterPlus R packages. The survival analysis of two subgroups was performed using the Kaplan–Meier survival curves with the log-rank algorithm utilizing R package “survival.”

### 2.3. Functional Enrichment Analysis

DEGs with |log2 − fold change| ≥ 1 and adjusted *P* value of <0.05 between the two groups were obtained using packages (limma, ggplot2) within the R software (v4.2.0) [34]. Gene Ontology (GO) enrichment analysis, Kyoto Encyclopedia of Genes and Genomes (KEGG) pathway analysis, and Gene Set Enrichment Analysis (GSEA) were performed using the R package cluster profiles. The GO analysis had three parts, which were as follows: biological process, cellular component, and molecular function. The cutoff *q* and *P* values were<0.05.

### 2.4. Immune Property Analysis in the Two ICD Subgroups

Tumor stromal and immune cells in SKCM were estimated using the ESTIMATE algorithm. R package “estimate” was performed to calculate stromal, immune, ESTIMATE scores, and tumor purity. To identify the immune characteristics of BRAF V600E WT SKCM samples, we performed the CIBERSORT (https://cibersort.stanford.edu/) algorithm to determine the relative levels of the 22 immune cells. Subsequently, the differential expression analysis of eight immune-checkpoint-relevant genes and 24 human leukocyte antigen (HLA) genes was performed between the two groups using R packages (limma, plyr, reshape2, ggplot2, and ggpubr).

### 2.5. Prognostic Modeling

The BRAF V600E WT dataset was assigned as the training set and the BRAF V600E-mutant dataset as the testing set. We utilized univariate Cox regression analysis for determining prognostic-related ICD genes in these two datasets. Reducing the candidate genes was conducted using the LASSO Cox regression model in the glmnet R package [35]. A *P* value of <0.05 was considered statistically significant. A multivariate Cox regression analysis was performed, and a risk-scoring equation was established according to the standardized regression coefficients of factors. According to the median value of the risk score, the SKCM samples were classified as high or low risk. The Kaplan–Meier survival curves were developed using the log-rank algorithm. Independent prognostic value of the risk core in SKCM was investigated via univariate and multivariate Cox analyses. We performed the time-dependent receiver operating characteristic (ROC) curves to determine how accurate the risk model was at predicting the prognosis of SKCM. Subsequently, a nomogram was constructed to predict the survival of patients using the R package “rms.” The calibration diagram was a common parameter used to assess the accuracy of nomograms. The CIBERSORT algorithm was applied to assess the infiltration of immune cells in the tumor microenvironment (TME) and to make a comparison between the risk score and the immune infiltration levels [36].

### 2.6. Prediction of Response to Immunotherapy

Tumor immune dysfunction and exclusion (TIDE) (http://tide.dfci.harvard.edu/) was performed for prioritization function and survival correlation function [37]. This is a computational approach that mimics two tumor immune evasion mechanisms for predicting immune checkpoint blockade response. In our study, TIDE was utilized to evaluate the predictive value of the ICD risk signature based on the potential clinical efficacy of immunotherapy.

## 3. Results

### 3.1. Typing and Grouping of Genes Associated with ICD and Its Associated Prognosis

**Figure 1:**
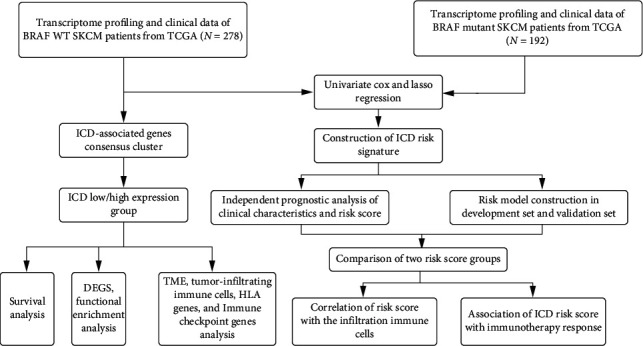
A schematic flow chart of the study.

**Figure 2:**
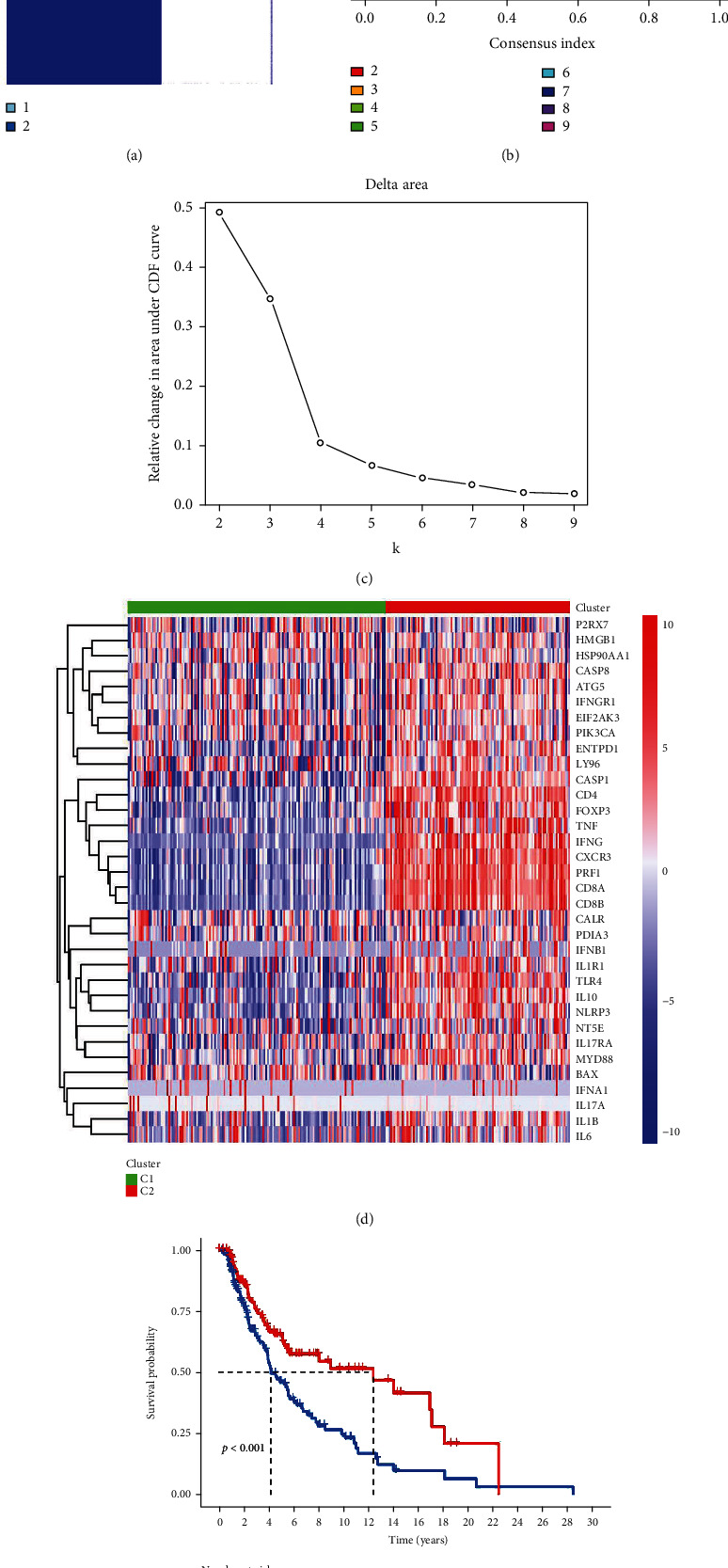
Identification of ICD-related subgroups using consensus clustering in BRAF V600E WT SKCM cohort. (a) Heatmap depicts consensus clustering solution (*k* = 2) for 34 genes in 470 SKCM samples. (b) Cumulative distribution function (CDF) curve for *k* = 2–9. (c) Delta area curve of consensus clustering indicates the relative change in area under the cumulative distribution function (CDF) curve for *k* = 2 to 10. (d) Heatmap of 34 ICD-related gene expressions in cluster C1 and cluster C2. Red represents high expression and blue represents low expression. (e) The Kaplan-Meier survival curve analysis reveals that the OS rate of patients in ICD-high group is higher than those in ICD-low group.

Figure 1 presents the flow process diagram of our study. To effectively differentiate patients with BRAF V600E WT SKCM in TCGA cohort according to ICD-associated genes, cellular component analysis of all samples was performed. A consensus matrix (*k* = 2) was selected as the criterion for disease type, and the samples were classified as clusters C1 or C2 (Figures 2(a)–2(c)) (Supplemental document 1_data of Figures 2A–2C). Clusters C2 and C1 were identified as ICD-high and ICD-low, respectively, based on the expression levels of ICD-related genes (Figure 2(d)) (Supplemental document 2_data of Figures 2A–2C). The abscissa depicted the name of samples. Meanwhile, the ordinate depicted the level of the ICD-related genes. Thus, the low expression group comprised the cluster C1 isoform with 159 patients, and the high expression group included the cluster C2 isoform with 114 patients (Supplemental document 2_data of Figures 2A–2C. The Kaplan–Meier curve revealed that the ICD-low subgroup had a poorer prognosis than the ICD-high subgroup (Figure 2(e)).

### 3.2. Functional Enrichment Analysis of the Two Subgroups

**Figure 3:**
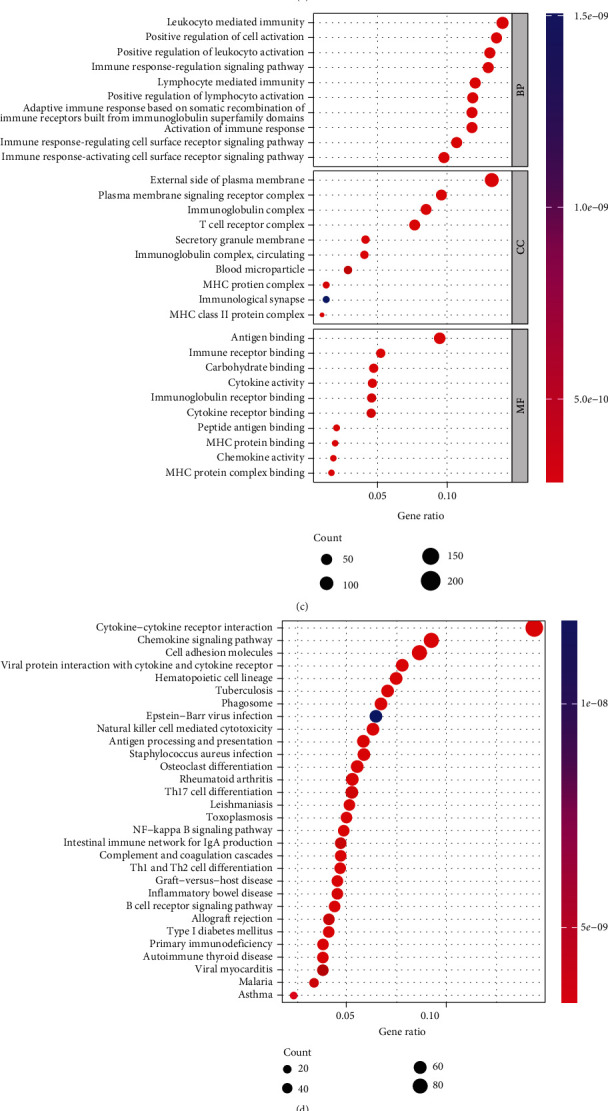
Identification of differentially expressed genes (DEGs) and underlying signal pathways in different subgroups of BRAF V600E WT SKCM cohort. (a) Volcano plot presents the distribution of DEGs quantified between ICD-high and ICD-low subgroups with threshold of |log2 Fold change| > 1 and *P* < 0.05. The red dot indicates the upregulated genes, whereas the green dot denotes the downregulated genes. (b) Heatmap shows the DEGs expression in different subgroups. (c) Functional analysis based on the DEGs between the two subgroups using GO enrichment analysis. (d) Functional analysis based on the DEGs between the two subgroups using KEGG pathway analysis. (e, f) GSEA GO identifies the related signal pathways between ICD-high and ICD-low subgroups.

For the different survival rate in two subgroups, we evaluated the key DEGs and biological pathways in each subgroup to explore the molecular mechanism in the regulation of prognosis. In total, 2476 DEGs were identified between the ICD-high and ICD-low groups (Figures 3(a) and 3(b)). Results showed that the DEGs were enriched in the immune activation and immune response pathways (Figure 3(c)). KEGG analysis resulted mainly in cytokine–cytokine receptor interaction (Figure 3(d)). GSEA was performed to determine the potential diversity of signaling pathways activated in the two subgroups. Results showed that ICD-high group was involved in immunoreactive pathways, such as immunoglobulin complex, immunoglobulin complex circulating, T-cell receptor complex, antigen binding, and immunoglobulin receptor binding (Figure 3(e)). However, involvement in epidermal cell differentiation, keratinization, keratinocyte differentiation, regulation of water loss in the skin, and cornified envelope was more significant in the ICD-low group than in the ICD-high group (Figure 3(f)). An association was observed between a high ICD and an immune-active microenvironment.

### 3.3. Tumor Immune Environment Properties in the Two ICD-Associated Groups

**Figure 4:**
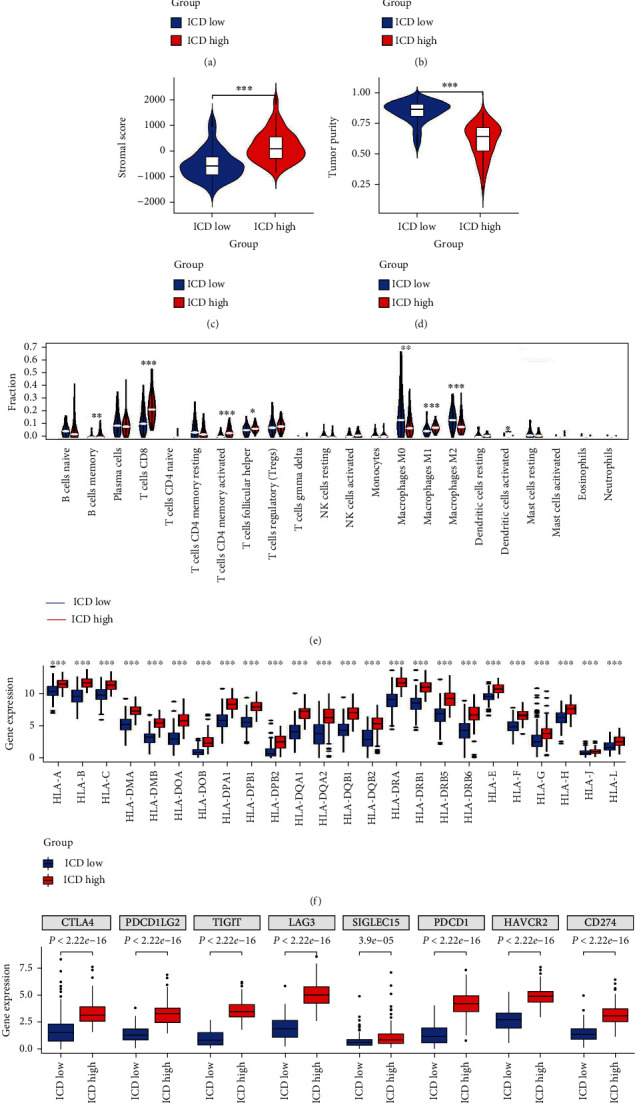
Tumor microenvironment and TIICs in the ICD-associated subgroups of BRAF V600E WT SKCM cohort. (a–d) Violin plots display the median, and quartile estimations for each.ESTIMATE score (a), immune score (b), stromal score (c), and tumor purity (d) in two subgroups. ^∗^*P* < 0.05, ^∗∗^*P* < 0.01, and ^∗∗∗^*P* < 0.001. (e) Violin plot visualizes the distinction of 22 immune infiltration cells between ICD-high and ICD-low subgroups. (f, g) Box plots present differential expression of multiple HLA genes (f), and immune checkpoints genes (g) between ICD-high and ICD-low subtypes. ^∗^*P* < 0.05, ^∗∗^*P* < 0.01, ^∗∗∗^*P* < 0.001, and ^∗∗∗∗^*P* < 0.0001.

Previous studies have found that ICD can activate the tumor's immune microenvironment. We dissected the composition of the TME in the two subgroups. Results showed that the ICD-high expression group had higher immune, stromal, and estimate scores than the ICD-low expression group (Figures 4(a)–4(c)). Further, the tumor purity of the ICD-high expression group was lower than that of the ICD-low expression group (Figure 4(d)). In addition, the violin plot showed the different proportions of 22 tumor-infiltrating cells between different groups based on the CIBERSORT algorithm (Figure 4(e)). Immune-boosting cells, such as CD8 T cells, CD4 memory-activated T cells, follicular helper T cells, macrophages M1, and monocyte-activated NK cells, were more abundant in the ICD-high group than in the ICD-low group (Figure 4(e)). HLA is produced by the main human histocompatibility complex, which regulates the body's immune response. The ICD-high group had a higher gene expression level of the 24 HLA antigens than the ICD-low group (Figure 4(f)). Due to the significant clinical progress of immune checkpoint inhibitors (ICI) in SKCM, the expression level of eight ICI-relevant genes in the two groups was evaluated. According to Figure 4(g), a high ICD high was associated with an increased ICI expression.

### 3.4. Prognostic Signature of ICD-Associated Genes

**Figure 5:**
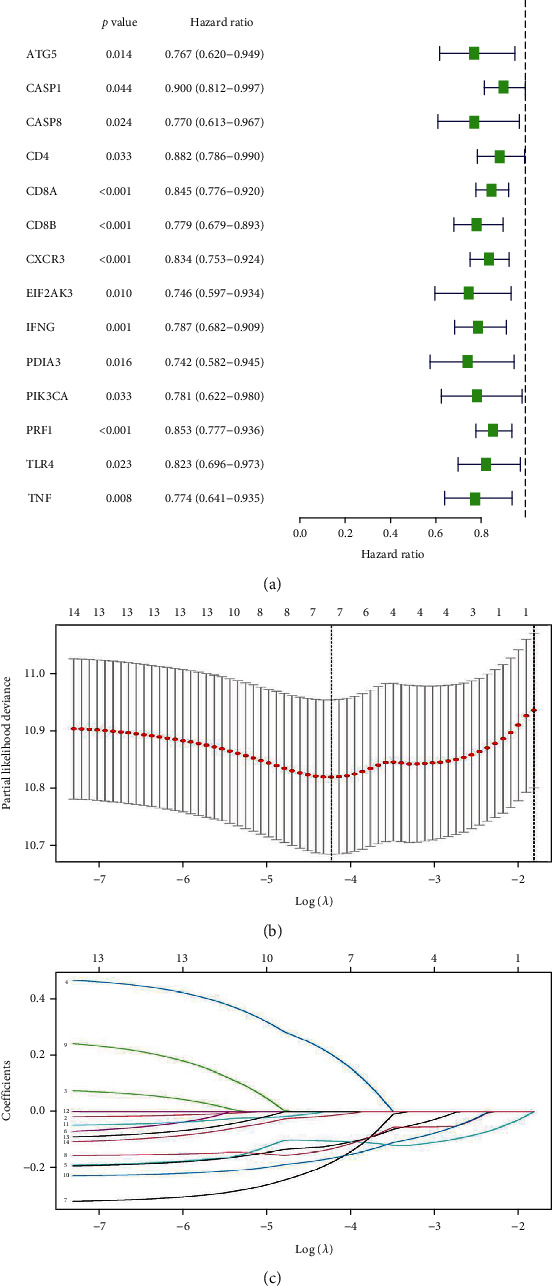
Identification of prognostic ICD-related genes in BRAF V600E WT and mutant SKCM. (a) Univariate Cox regression analysis suggests that 14 ICD-related genes are associated with OS in SKCM. (b, c) Least absolute shrinkage and selection operator analysis (LASSO) shows the minimal lambda and coefficients of prognostic ICD-related genes.

In order to evaluate the prognostic signature of patients with CM, 34 ICD genes were retrived. 14 ICD-related genes association with the OS were screened via Cox univariate analysis (Figure 5(a)). Second, LASSO cox regression analysis was performed to reduce multicollinearity, thereby narrowing the number of ICD-related genes down to 7 (Figures 5(b) and 5(c)).

**Figure 6:**
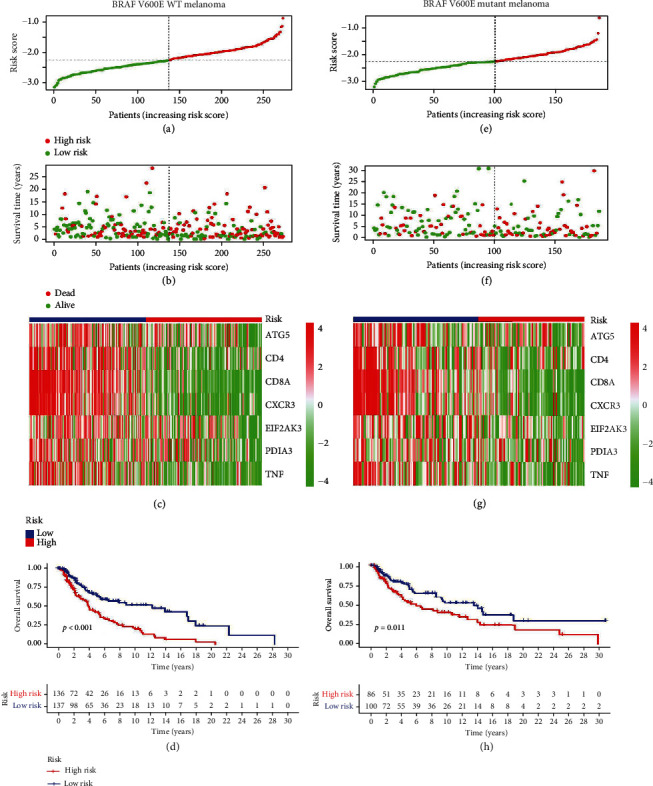
Construction and validation of the ICD-related genes risk model in BRAF V600E WT and mutant SKCM. The distribution of risk score calculated by ICD-related genes prognostic signature and the scatter dot plot shows the association of risk score and survival time in BRAF V600E WT SKCM cohort (a–c) and BRAF V600E mutant SKCM cohort (e–g). The Kaplan-Meier survival curve displays the OS rate of patients with SKCM in low- and high-risk group in BRAF V600E WT cohort (d) and BRAF V600E mutant cohort (h).

A risk scoring system was established based on the seven ICD-related genes for predicting OS in patients with the BRAF V600E WT melanoma cohort (Figures 6(a)–6(d)). We calculated the risk score for each patient based on individualized ICD-related signature levels. The risk score was calculated as follows: (−0.120643431584661) × ATG5 + (0.203633786353922) × CD4 + (−0.104053110194778) × CD8A + (−0.180389376548664) × CXCR3 + (−0.13208348313494) × EIF2AK3 + (−0.164918261157723) × PDIA3 + (−0.0135828017840705) × TNF. The median risk score was used to categorize patients into the high- and low-risk groups (Figure 6(a)). Then, we developed the risk curves and scatter plots to indicate the risk score and survival status of each patient with SKCM. The risk coefficient and the number of dead statuses in the high-risk group were higher than those in the low-risk group (Figure 6(b)). Heatmap visualization results revealed the expression differences of seven ICD-related genes in the low- and high-risk groups. The low-risk groups had a higher expression of the seven genes (Figure 6(c)). Kaplan–Meier analysis revealed a sound OS in the low-risk group in the BRAF V600E WT SKCM cohort (*P* < 0.05, Figure 6(d)).

This model was also applied to the BRAF V600E mutant SKCM cohort (Figures 6(e)–6(g)). In the low-risk group, there were significantly more alive statuses, as seen in Figure 6(f). In the BRAF V600E mutant melanoma, the result further confirmed that poor OS was correlated with a high-risk score (Figure 6(h)). The prognosis of both types of SKCM could be accurately predicted using the prognostic signature of seven ICD-related genes based on these findings.

### 3.5. Verification of the Risk Model

**Figure 7:**
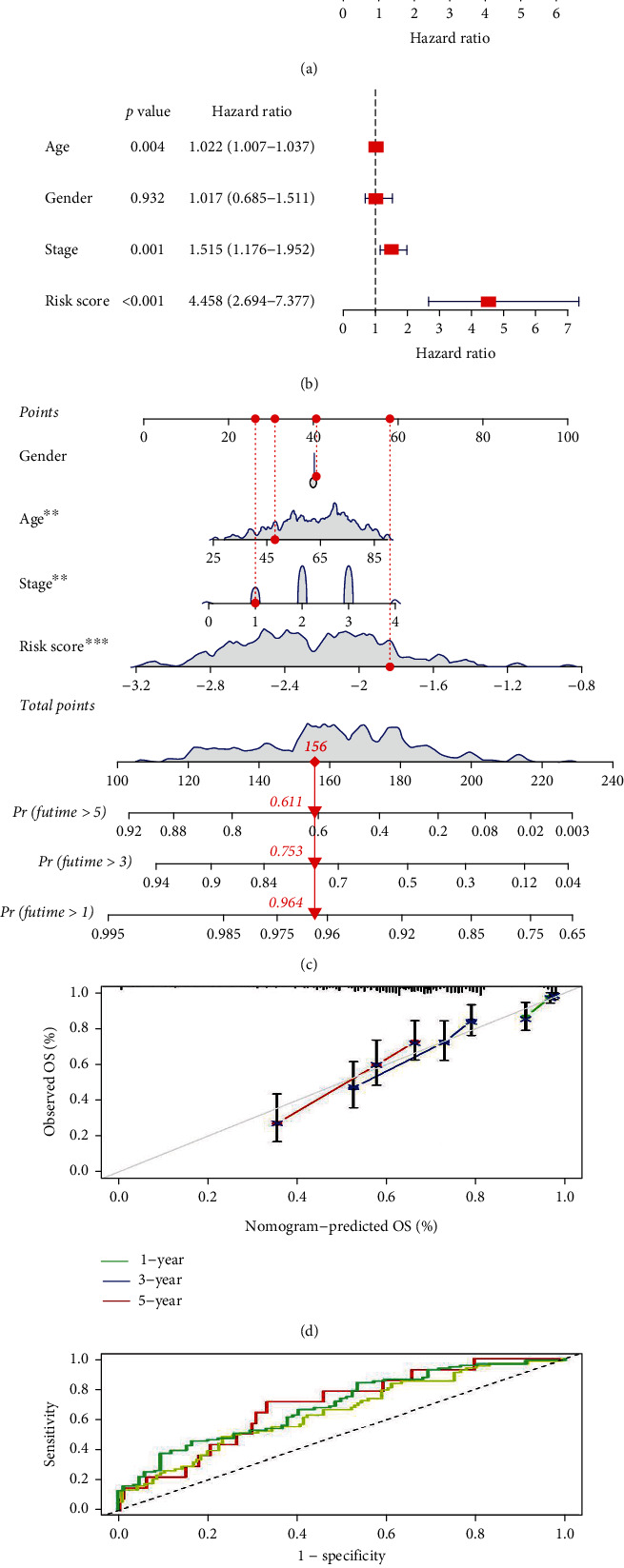
Independent prognostic analysis of clinical characteristics and risk score in BRAF V600E WT SKCM cohort. (a) Univariate Cox regression analysis suggests a clear association between OS rate and clinical characteristics including age, gender, stage, and the risk score. (b) Multivariate Cox regression analysis indicates that age, stage, and risk score are independent prognostic indicators for SKCM. (c) Nomogram construction of risk score and linicopathological characteristics to predict the 1-, 3-, and 5-years OS rate of SKCM patients. (d) Calibration curve shows the accuracy between predictive capacity and actual OS rate of 1-, 3-, and 5-years. (e) Time-dependent ROC curve shows the AUC at 1-, 3-, and 5-years.

To study the impact of ICD-related gene score on clinical characteristics, univariate Cox analysis was utilized to investigate that age, stage, and risk score were associated with OS in patients with BRAF WT SKCM. The hazard ratios (HR) were 1.019 (95% confidence interval [CI]: 1.004-1.034), 4.081 (95% CI: 2.495–6.674), and 1.436 (95% CI: 1.136–1.815) (Figure 7(a)), respectively (all *P* values <0.05). Further multifactorial Cox regression analysis showed that the risk score was an independent risk factor of OS (hazard ratio = 4.458, 95% CI: 2.694–7.377, *P* < 0.001) (Figure 7(b)). In addition, a nomogram integrating gender, age, tumor grade, and risk score was established to predict the 1-, 3-, and 5-year survival probability of patients with BRAF V600E WT SKCM (Figure 7(c)). The calibration curves validated that the predictive ability of the nomogram was considerably good (Figure 7(d)). According to the time-dependent ROC, the areas under the curve (AUCs) for 1-, 3-, and 5-year survival were 0.693, 0.650, and 0.695, respectively (Figure 7(e)).

### 3.6. Immune Cell Correlation Analysis and Immunotherapy Analysis with ICD Risk Scores

**Figure 8:**
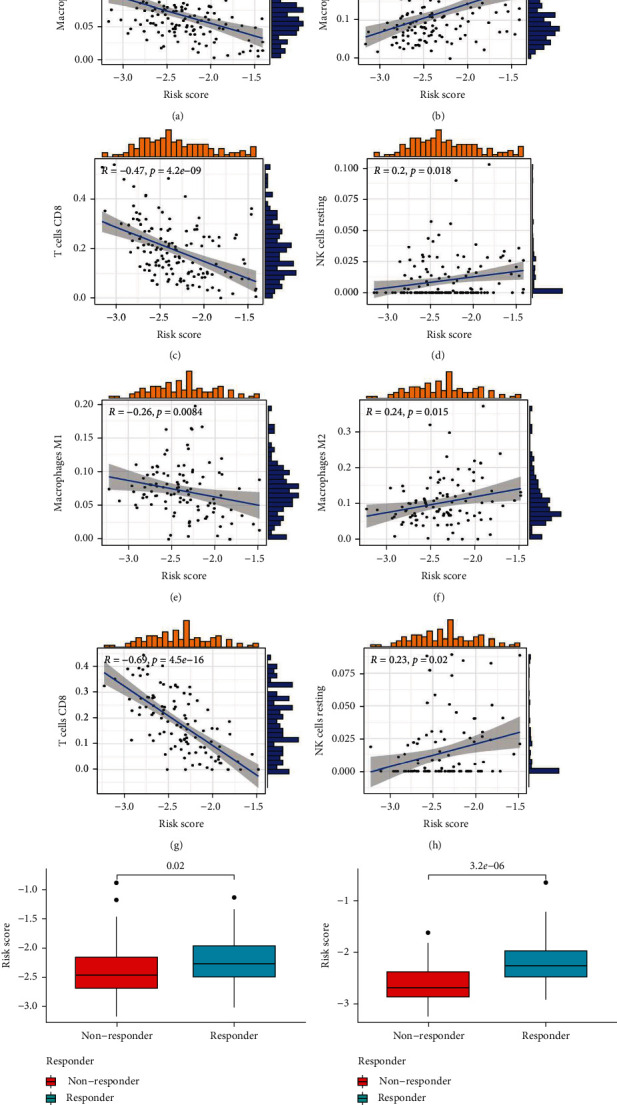
The association of ICD risk signature with tumor microenvironment. Scatter plots display the association of risk score with the expression level of infiltration of immune cells in BRAF V600E WT SKCM cohort (a–d) and BRAF V600E mutant SKCM cohort (e–h). Box plot presents the correlation of risk score with immunotherapy response in BRAF V600E WT cohort (i) and BRAF V600E mutant cohort (j).

Considering the importance of ICD in antitumor immune responses, the association between the ICD risk score and the tumor microenvironment was cautiously examined. As shown in the BRAF V600E WT SKCM cohort, the risk score was negatively associated with immune-boosting cells (CD8 T cells, macrophages M1; Figures 8(a)–8(c)) and positively associated with immunosuppressive cells (resting NK cell, macrophage M2; Figures 8(b) and 8(d)). The BRAF V600E WT SKCM cohort and the BRAF V600E-mutant SKCM had similar outcomes (Figures 8(e)–8(h). These cells were formerly accepted in cancer immunosurveillance [38], autoimmunity [39], and immunosuppression [40]. According to these studies, a high-risk score might be associated with the immunosuppressive microenvironment in SKCM. In addition, the current study showed a negative association between risk scores and immunotherapy response. The immunotherapy response group had a lower risk score for BRAF V600E WT and BRAF V600E-mutant SKCM (Figures 8(i) and 8(j)). Therefore, immunotherapy may be more beneficial to patients with low-risk scores.

## 4. Discussion

The current study may be summarized as follows. First, in patients with BRAF V600E WT SKCM, compared with the ICD-low group, the ICD-high group had significantly more immune-enhanced cell infiltration, immune-activated signaling pathway aggregation, tumor microenvironment effects, and a better clinical prognosis. Second, a prognosis model based on ICD-related genes is feasible for assessing the prognosis of both BRAF V600E WT and mutant SKCM, its influence on the tumor microenvironment, and immunotherapy response. These results could be used to explore novel prognosis biomarkers or construct models for case selection for immunotherapy in patients with SKCM who have different BRAF V600E statuses.

Melanoma is an immunogenic tumor, and immunotherapy has been extensively evaluated in melanoma. Although immunotherapy is associated with significantly better melanoma outcomes, its response rate is limited [41, 42]. Therefore, in patients with BRAF V600E WT SKCM, selecting a population suitable for immunotherapy is important in improving survival. In a cohort with BRAF V600E WT SKCM, the two ICD-related subgroups were established, and the OS and tumor microenvironment of these subgroups were compared. ICD-high might be associated with the active tumor immune environment and a favorable OS. BRAF V600E mutations are prevalent in melanoma. Small-molecule inhibitors of BRAF (e.g., vemurafenib, dacarbazine, and encorafenib) are significantly associated with prolonged OS in patients with BRAF V600E-mutant SKCM [43–45]. However, due to drug resistance and the emergence of toxic reactions for targeted drugs, combined therapy with immunotherapy has been further explored. Hence, risk stratification is essential in improving survival in patients with BRAF V600E-mutant SKCM. Thus, a risk model was constructed to predict the prognosis of BRAF V600E-mutant SKCM, and the difference between the immune microenvironment and immunotherapy response in the two risk subgroups was explored.

ICD is a regulated type of cell death that activates adaptive immunity and promotes long-term immune memory. Immune checkpoint inhibitors (ICIs) are the most effective therapy against inflammatory tumors with antitumor T cells and immune effector cytokines, and these tumors are known as hot tumors [46]. ICD can be induced in cancer cells using chemotherapy [47], hypericin-based photodynamic therapy [48], high hydrostatic pressure [49], and irradiation [50, 51]. This may help create an inflamed, immunogenic tumor environment that transforms the cold TME into a hot TME, thereby addressing therapeutic resistance in ICI. When we predicted the efficacy of immunotherapy treatment for SKCM patients using TIDE software, we found that TIDE scores were significantly higher in the responder subgroups than in nonresponder subgroups (*p* < 0.05, Figures 8(i) and 8(j)). This finding demonstrates the IRGs signature has the potential to predict immunotherapy efficacy.

In the current study, compared with ICD-low, ICD-high was associated with a higher proportion of immune boosting cells and a lower proportion of immunosuppressive cells. Abundant CD8+ T cell expansion is observed in inflammatory metastatic melanoma, and it drives the upregulation of PD-L1 and Treg in the tumor microenvironment [52]. Furthermore, CD8+ T cell expansion is correlated with a better response to anti-PD1 immunotherapy in patients with cancer [53]. The macrophage M1 advantage of the ICD-high group is consistent with its attested function in tumor development. Meanwhile, the enrichment of macrophages M2 in the ICD-low group reflects the promotion of tumor growth and transformation [54]. Activated dendritic cells are considered a formidable independent prognostic factor in antitumor immune response [55]. The results in our study showed that risk score was proportional to resting NK cells. A report shows that human melanoma cells with characteristics of cancer stem cells will be killed by NK cells [56]. Therefore, these studies support the notion that the stratification of patients with SKCM according to ICD expression can differentiate patients with a strong immune activation state from those with a poor immune microenvironment.

In our study, we explored a coexpression network of IRGs by R. The risk model was created by seven IRGs. And the *ATG5*, *CXCR3,* and *CD8A* genes were prognostic ICD-related genes in SKCM. *ATG5* (autophagy-related 5), which is a key linker protein in the autophagy pathway, is indispensable in both classical and nonclassical autophagy [57]. Recent studies have shown that ATG5 regulates the immune system and interacts with apoptosis [58, 59]. ATG5-dependent autophagy can regulate macrophage polarization [60] and cause the expulsion of lipopolysaccharide-stimulated mitochondrial contents, which leads to an inflammatory response in immune cells [61]. ATG5 indirectly activates lymphocytes by promoting interactions between T or B cells and antigen-presenting cells via autophagy [62]. Furthermore, it can bind in the form of a complex to the caspase recruitment domain of the melanoma differentiation-related gene 5 to inhibit the production of type I interferon and secretion [63], thereby participating in the adaptive immune regulation of the body. The *ATG5* expression in the high-risk group was lower in our risk model. Whether this may cause the body to have a weaker adaptive immune modulatory ability and lead to poorer immunotherapy response and prognosis should be further evaluated. *CXCR3*, a common receptor of chemokines *CXCL9*, *CXCL10*, and *CXCL11*, has a chemotactic effect. Further, it participates in regulating the differentiation and development of memory and effector T cells [64]. A previous study showed that CXCR3 is a biomarker of response for checkpoint inhibitor therapy in skin cancer [65]. This partly explains that the CXCR3 expression level is correlated with a lower risk score and a better prognosis. We found that the low *CD8A* gene expression level was associated with poor prognosis. *CD8A* is associated with T cell function and immune signaling. Ulloa-Montoya et al. [66] revealed that *CD8A* was associated with clinical benefits in patients with melanoma. Here, we confirmed that the risk scores could be considered a reliable and independent prognostic biomarker for BRAF V600E WT SKCM patients. The ROC values for 1-, 3-, and 5-year survival showed that the risk model had good stability and accuracy. When we utilized the risk score and other clinicopathological parameters to develop a nomogram, the results indicated that the AUC values of ROC curves were significantly higher compared to the single-factor values (age, risk score, gender, and stage). The calibration diagram indicated that the predicted curve was closer to the ideal curve, proving that the developed nomogram could improve the predictive ability and accuracy to BRAF V600E WT SKCM patients. So the 7-gene signature panel developed in our study can soundly predict prognosis for melanoma.

Our results showed that different pathways, such as adaptive immune response based on leukocyte-mediated immunity, antigen binding, cytokine−cytokine receptor interaction, chemokine signaling pathway, immunoglobulin complex, and antigen processing and presentation, were enriched in the ICD-high group. These are immune activity-related pathways, which can better respond to immunotherapy.

The current clinical trial uses combined chemotherapeutic drugs and Food and Drug Administration-approved ICI targeting either PD-1 or PD-L1 [67, 68]. This therapy is aimed at initiating ICD after chemotherapy and turn immune cold tumors into hot ones, thereby making the tumor respond to ICI. TME plays an important role in immunotherapy response [69]. Here, the ICD-high expression patients showed higher ESTIMATEScore, ImmuneScore, and StromalScore, and lower tumor purity values than ICD-low expression patients, representing more immune or stromal cellular components in the TME. Our study found that several immune-related genes, in addition to the currently known *CD274*, *CTLA-4*, and *LAG3,* might be targeted. The elevated expressions of *HAVCR2*, *TIGIT*, *PDCD1*, *PDCD1LG2*, and *SIGLEC15* were evident in the ICD-high group, which hints at an alternate immunosuppressive direction. This was originally proposed in the cutaneous melanoma cell line [70]. These markers may also provide better treatment outcomes in patients with ICD-high.

The current study had certain limitations. First, the samples of the database all came from the public data and the size was limited. This may lead to some potential errors or biases in this model. Larger sample size or even prospective studies are needed to be conducted to confirm these findings. Second, functional and mechanistic research should be conducted to better understand the role of ICD-related genes in BRAF V600E WT and mutant SKCM.

## 5. Conclusions

The ICD-related subgroups of patients with BRAF V600E WT SKCM have different prognoses and immune landscapes. These results may provide a reference for the stratified management of patients when choosing immunotherapy strategies. We screened out the ICD genes associated with the prognosis of BRAF V600E WT and mutant SKCM. ICD-related prognostic signatures were established and validated. Novel ICD-related features (*ATG5*, *CD4*, *CD8A*, *CXCR3*, *EIF2AK3*, *PDIA3*, and *TNF*) are associated with the immune microenvironment and immunotherapy, which may be valuable prognostic markers in patients with BRAF V600E WT and mutant SKCM.

## Data Availability

All data generated or analyzed during this study are included in this published article.

## Supplementary Materials

Supplementary 1 Supplemental document 1_data of Figures 2A–2C. To effectively differentiate patients with BRAF V600E WT SKCM in TCGA cohort according to ICD-associated genes, cellular component analysis of all samples was performed. A consensus matrix (*k* = 2) was selected as the criterion for disease type, and the samples were classified as clusters C1 or C2.

Supplementary 2 Supplemental document 2_data of Figures 2A–2C. Clusters C2 and C1 were identified as ICD-high and ICD-low, respectively, based on the expression levels of ICD-related genes.

Supplementary 3 Supplementary Table S1. Clinical characteristics of SKCM patients from TCGA data in the study.
