# Supplementary material for: An Immunogenic Cell Death-Related Gene Signature Reflects Immune Landscape and Predicts Prognosis in Melanoma Independently of BRAF V600E Status
Source: Biomed Res Int. 2023 Jan 16;2023:1189022. doi: 10.1155/2023/1189022 (PMC9871414; doi:10.1155/2023/1189022)
Supplement: Supplementary 3 — Supplementary Table S1. Clinical characteristics of SKCM patients from TCGA data in the study. [file 1189022.f3.pdf]

Table S1. Clinical characteristics of SKCM patients from TCGA data in the study.

Variable	No. of samples in TCGA
Gender	
Male/Female	290/180
Age at diagnosis	
<=65/>65/unknown	298/164/8
Clinical stage	
0/I/II/III/IV/unknown	7/77/140/171/23/52
BRAF V600E status	
wild-type	278
mutant	192

SKCM, skin cutaneous melanoma; TCGA, the Cancer Genome Atlas.
